# Chancre Redux after Salvarsan

**Published:** 1914-04

**Authors:** 


CHANCRE REDUX AFTER SALVARSAN. W. Dubreuilh and G. Petges, *Jour. de Med. de Bordeaux,* August 24, 1913, report a case, the differentiation from reinfection being the lack of secondary manifestations, the new chancre appearing on the old site, 20 months after the first infection. They also report similar cases (2) after mercurial treatment.

